# Laboratory Relationships between Adult Lifetime Reproductive Success and Fitness Surrogates in a *Drosophila littoralis* Population

**DOI:** 10.1371/journal.pone.0024560

**Published:** 2011-09-09

**Authors:** Nina Pekkala, Janne S. Kotiaho, Mikael Puurtinen

**Affiliations:** 1 Centre of Excellence in Evolutionary Research, Department of Biological and Environmental Science, University of Jyväskylä, Jyväskylä, Finland; 2 Natural History Museum, University of Jyväskylä, Jyväskylä, Finland; The University of Queensland, Australia

## Abstract

The difficulties in measuring total fitness of individuals necessitate the use of fitness surrogates in ecological and evolutionary studies. These surrogates can be different components of fitness (e.g. survival or fecundity), or proxies more uncertainly related to fitness (e.g. body size or growth rate). Ideally, fitness would be measured over the lifetime of individuals; however, more convenient short-time measures are often used. Adult lifetime reproductive success (adult LRS) is closely related to the total fitness of individuals, but it is difficult to measure and rarely included in fitness estimation in experimental studies. We explored phenotypic correlations between female adult LRS and various commonly used fitness components and proxies in a recently founded laboratory population of *Drosophila littoralis*. Noting that survival is usually higher in laboratory conditions than in nature, we also calculated adjusted adult LRS measures that give more weight to early reproduction. The lifetime measures of fecundity, longevity, and offspring viability were all relatively highly correlated with adult LRS. However, correlations with short-time measures of fecundity and offspring production varied greatly depending on the time of measurement, and the optimal time for measurement was different for unadjusted compared to adjusted adult LRS measures. Correlations between size measures and adult LRS varied from weak to modest, leg size and female weight having the highest correlations. Our results stress the importance of well-founded choice of fitness surrogates in empirical research.

## Introduction

Fitness can be defined as a property of a phenotype (or genotype) that predicts its representation in future generations [1]–[6]. Evolutionary biologists often seek to measure the fitness of particular phenotypes (or genotypes) in order to understand and predict changes in the constitution of populations. Measuring fitness is not a simple task, and the best measure of fitness can differ depending on the biology of the study system. Particularly, the strength of genotype-by-environment interactions on fitness [3], [4] and, in species with overlapping generations, the rate of reproduction [3], [7]–[9], need to be considered when measuring fitness. For species with non-overlapping generations, and for populations at constant population size, the best measure of individual fitness is the lifetime reproductive success, i.e. the number of viable zygotes produced over the whole life-cycle of the individual [3], [6].

Measuring the total fitness of individuals is often unfeasibly demanding. Instead, researchers use various fitness surrogates, traits that are thought to reflect fitness and are relatively easy to measure. Fitness components, such as fecundity and survival, are by necessity related to fitness [6], and are thus often preferred as fitness surrogates in empirical studies [10]–[13]. However, these traits are seldom measured over the whole lifetime of individuals, but only over a restricted time frame that is most feasible for the study system. Besides different components of fitness, morphological and behavioral traits such as body size, growth rate, dominance, and mating success, are often used as surrogates of fitness [12], [14], [15]. The association between these so called fitness proxies and total fitness of individuals is more uncertain than that between fitness components and total fitness, but they are often measured due to their convenience [6]. Using any fitness surrogate without empirical knowledge about the true relationship of the surrogate and total fitness may lead to erroneous conclusions.

Adult lifetime reproductive success (adult LRS) is likely to be closely related to total fitness of individuals, as it combines several components and proxies of fitness (longevity, fecundity, offspring viability, mating success, etc.). Brommer et al. [16] have shown adult LRS to be a good predictor of long-term genetic contribution to the population in natural populations of two bird species. Adult LRS is, however, difficult to measure and therefore rarely included in fitness estimation in experimental studies.

To evaluate the reliability of various commonly used fitness surrogates, we explored phenotypic correlations between adult LRS, measured as the total number of offspring produced over the adult lifetime of individual females, and various morphological and life history traits, in a recently founded *Drosophila littoralis* laboratory population. *D. littoralis* is a boreal drosophilid belonging to the *D. virilis* species group. In northern Fennoscandia *D. littoralis* overwinters as adult, reproduces in the spring, and the next generation (summer generation) emerges before autumn [17]. The overwintered and summer generations overlap only slightly and only a small proportion of the summer generation reproduces during the ongoing summer [17]. The species is thus practically univoltine, with only slightly overlapping generations. However, noting that survival is usually higher in laboratory conditions than in nature where individuals are subject to predation and other hazards, we also calculated adjusted adult LRS measures that give more weight to early reproduction. Comparing the correlations of other fitness surrogates to adjusted and unadjusted adult LRS measures provides insight about the sensitivity of laboratory-derived fitness correlations to the higher mortality rates likely to exist in natural conditions. We explored phenotypic correlations between the adult LRS measures and fitness components measured over the lifetime of the females (longevity, lifetime fecundity, and lifetime egg-to-adult viability of offspring), fitness components measured over shorter periods throughout female life (short-time fecundity and short-time offspring production), and size measures often used as proxies of individual fitness (weight and several morphological measures of the females).

## Methods

### Ethics Statement

No permits are required for collecting flies by the Tourujoki River in Jyväskylä, Finland.

A laboratory population of *D. littoralis* was founded in spring 2006 from 157 males and 99 females collected from a natural population by the Tourujoki River in Jyväskylä, Finland. Thirty-four of the 99 females had been inseminated in the wild and produced fertile eggs after transfer to the lab. The rest of the females were mated randomly in the lab with the wild-caught males. Population size was increased to 419 breeding couples in F2. The parental flies were assigned randomly each generation, but inbreeding was reduced by preventing full-sib matings. In a sample of 20 individuals from F4, 11 out of 14 nuclear microsatellite loci were polymorphic [18]. In the polymorphic loci, the mean number of alleles was 6.8 and the mean observed heterozygosity was 0.55. The flies were kept in plastic vials (diameter 23.5 mm, height 75.0 mm) with malt-yeast medium [19], at 19°C and relative humidity of 60% with constant light. Generation length of the flies under these conditions is approximately 35 days.

In F3, we measured egg and offspring production for 84 females from 5 days after eclosion until death. Based on a pilot experiment, females don't produce eggs before this age (data not shown). All females were from different families. One female and one non-sib male (age 13–22 days from eclosion) were placed into a plastic vial with 8 ml of malt-yeast medium to mate and lay eggs. The couples were placed into a new vial every second day, which is sufficient to prevent crowding of the larvae (see Results). To make sure that female reproduction was not limited by male quality, the male was replaced with a new one (age 13–22 days) every second week, or immediately if it was found dead or if it escaped during handling. The number of eggs laid and the number of eclosing flies were counted from each vial. Mould or bacterial growth in vials was rare, and was not observed more often in vials with small number of eggs compared to vials with more eggs (personal observation).

We measured adult LRS as the number of eclosing offspring produced by an adult female over its lifetime. In optimal laboratory conditions with continuous availability of food and no predators the lifetime of *Drosophila* is much longer than in natural populations [20]. Thus, the lifetime reproductive success reached in laboratory conditions is rarely realized in nature. To further explore the possible consequences of higher mortality on the fitness surrogates, we calculated adjusted adult LRS measures by assuming additional values of daily mortality risk of 2, 4, 6, 8, 10, and 12% for the females. The offspring number in each vial was multiplied by the calculated survival probability to the specified age, and the adjusted offspring numbers of all the vials for each female were then summed together. The adjusted adult LRS thus equals the expected number of offspring a female with a certain reproductive history in laboratory would produce if there was some external factor, e.g. predation, inflicting a constant daily risk of mortality. Lifetime fecundity was measured as the number of eggs produced by an adult female over its lifetime. Offspring viability was measured for each female by dividing the total number of offspring produced (i.e. adult LRS) by the total number of eggs produced (i.e. lifetime fecundity; note that the fertilization rate of the eggs is not known).

The short-time estimates of offspring production and fecundity were calculated as sliding windows throughout female life. To be able to compare estimates based on time frames of different length, we used three different time frames: 2, 4 and 10 days. We also present the correlations of cumulative offspring production and cumulative fecundity with adult LRS. Comparing the correlations of the cumulative measures and the short-time measures may reveal the possible benefit of measuring offspring production or fecundity of individuals from sexual maturity to some specific age (i.e. cumulative measurement), versus measuring these traits only for a short period at a specific age.

**Figure 1:**
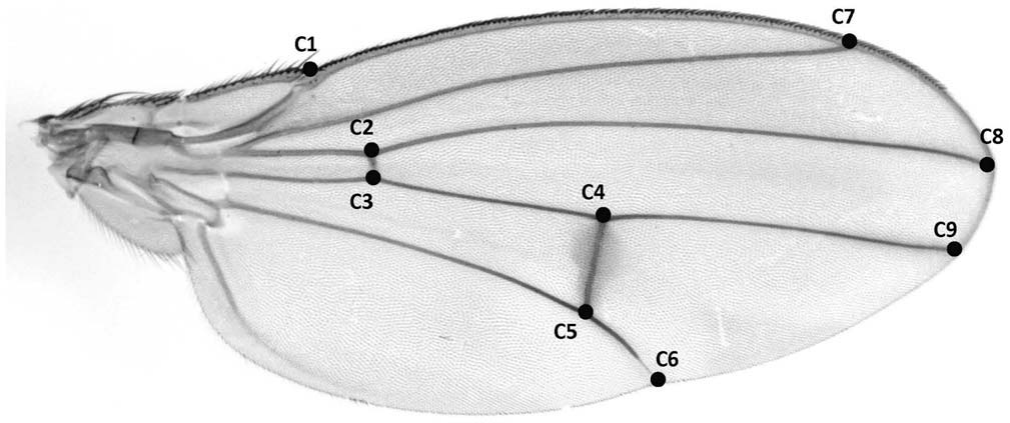
Landmarks for measurement of wing size (C1–C9).

The females were weighed in the beginning of the experiment (5 days after eclosion). After death, females were preserved in 70% ethanol. Several morphological measurements were taken from the preserved samples. The wings and hind legs of the flies were fixed on microscope slides and digitally photographed. Distance between nine cross points of the wing veins (Fig. 1) and length of femur, tibia, and the five segments of tarsus of hind legs were measured from the images. When measurements could be taken from both left and right wings or legs, we averaged the left and right measurements to get one estimate for each measurement for each fly. When only one measurement was possible due to damaged wings or legs (note that the flies had died of old age and were thus rather worn), the single available measurement was used. To obtain a single size component for wings and legs, we extracted the first principal component from the correlation matrix of the measurements. The size component for wing explained 78.5% of total variance with initial eigenvalue of 28.3. The size component for leg explained 50.3% of total variance with initial eigenvalue of 3.5. Length of thorax (longest distance between neck and the tip of scutellum measured from the side of the fly), length of scutellum (longest dorsoventral distance), and width of head (distance between eyes through ocelli) were measured using light microscope. Each fly was measured twice, and the mean of the two measurements was used in the analyses to reduce the measurement error.

Measurements done with light microscope had fairly low repeatabilities (thorax 0.85, scutellum 0.58 and head width 0.54). Using the average of two measurements of the same trait however reduces the measurement error. Measurements from wings and legs were taken from digital photographs and are less affected by measurement error. Calculating the repeatability from left and right measurements includes variance due to asymmetry, in addition to variance due to measurement error. Excluding the two most asymmetric individuals from analysis, distance measurements from left and right wings had average repeatability of 0.93, distance measurements from left and right legs had average repeatability of 0.60, and left and right measurements of tibia had repeatability 0.86. As pointed out above, these repeatabilities are affected by real within-individual asymmetry. As we used the average of the left and right-side measurements in all analysis, we were able to obtain individual estimates that were less affected by both asymmetry and measurement error.

Except for female longevity, all the variables were normally distributed (one-sample Kolmogorov-Smirnov test). Thus, we analyzed the parametric correlation coefficients between variables other than longevity, and both parametric and non-parametric correlation coefficients between longevity and the other variables. We corrected for multiple testing using the Benjamini & Hochberg correction for false discovery rate at 0.01 and 0.05 significance levels [21]. To examine the possible effect of crowding on offspring emergence, we tested the effect of number of eggs in a vial on egg-to-adult offspring viability with linear regression. All the analyses were performed with PASW Statistics 18.

## Results

**Figure 2:**
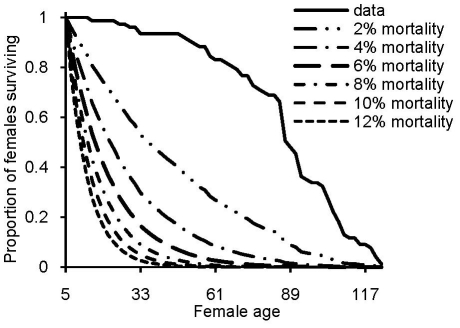
Female survival. Proportion of females surviving in the experiment (solid line), and expected survival probability with additional daily mortality risk of 2, 4, 6, 8, 10, and 12% (dashed lines) for different female ages (the dashed lines combine natural deaths with the additional mortality risk). Female age (in days) is scored according to the last day in a vial.

**Figure 3:**
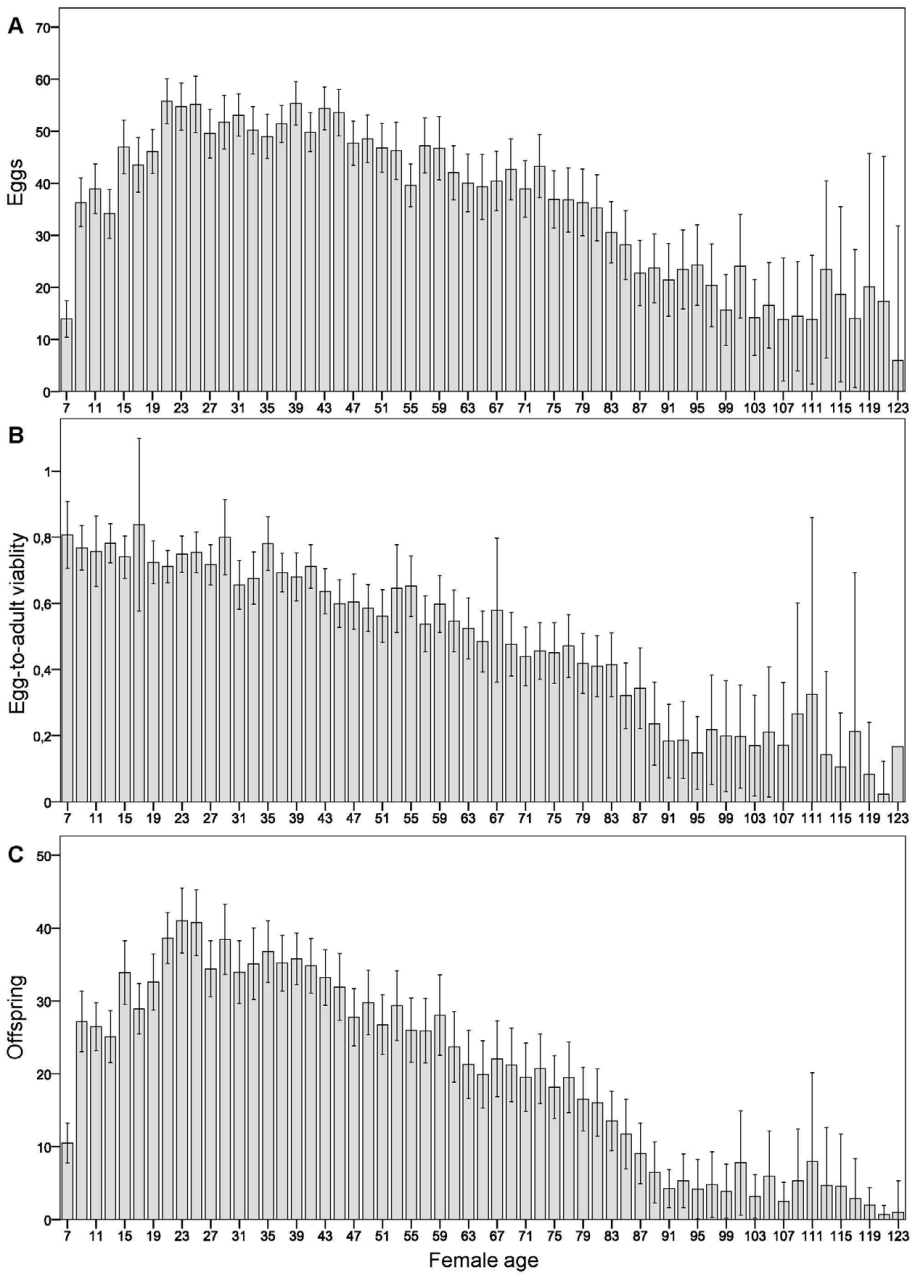
Effect of age on female reproduction. A) mean egg production, B) mean egg-to-adult viability of offspring, and C) mean offspring production, in relation to female age. Error bars indicate 95% confidence interval. Female age (in days) is scored according to the last day in a vial.

After removing females that accidentally escaped or died during handling, a total of 77 females remained in the analyses. The last female in the experiment was found dead at the age of 125 days (Fig. 2). Offspring production of the females decreased with aging, and this was due to combined effects of senescence on both female fecundity and on egg-to-adult viability of offspring (Fig. 3). Mean number of eggs laid by the females began to decrease approximately from the age of 45 days onwards. Mean egg-to-adult offspring viability showed a continuous decrease as the females aged. The peak in mean number of offspring produced was at the age of 21 to 25 days. Negative effect of senescence on female fecundity and offspring viability have been reported before e.g. in *D. melanogaster* [22], [23].

**Figure 4:**
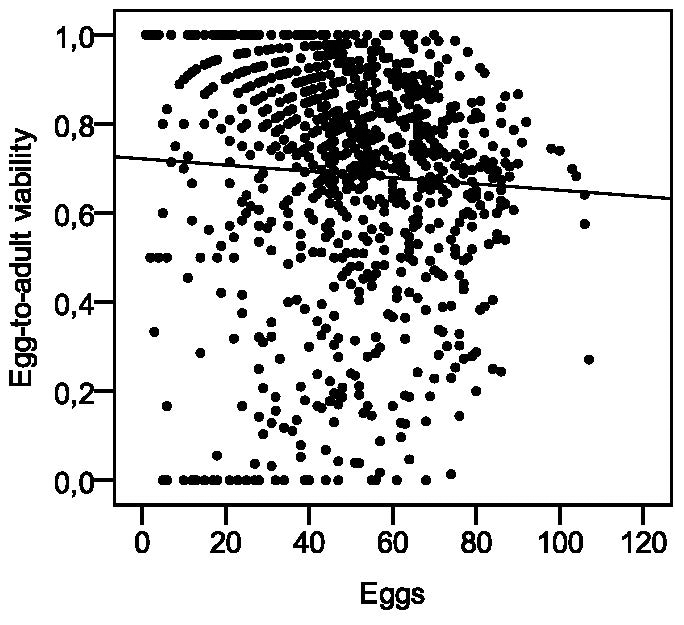
Egg-to-adult viability of offspring plotted against number of eggs in a vial. Number of eggs in a vial did not affect egg-to-adult viability of the offspring (linear regression of egg-to-adult viability on egg number: F_1,966_ = 2.997, R^2^ = 0.003, p = 0.084).

The possible effect of crowding on egg-to-adult viability of the offspring was tested for vials collected from the beginning of the experiment until the females were 35 days old, so that the effect of female aging on offspring viability could be minimized. Number of eggs in a vial did not affect egg-to-adult viability of the offspring (Fig. 4).

**Figure 5:**
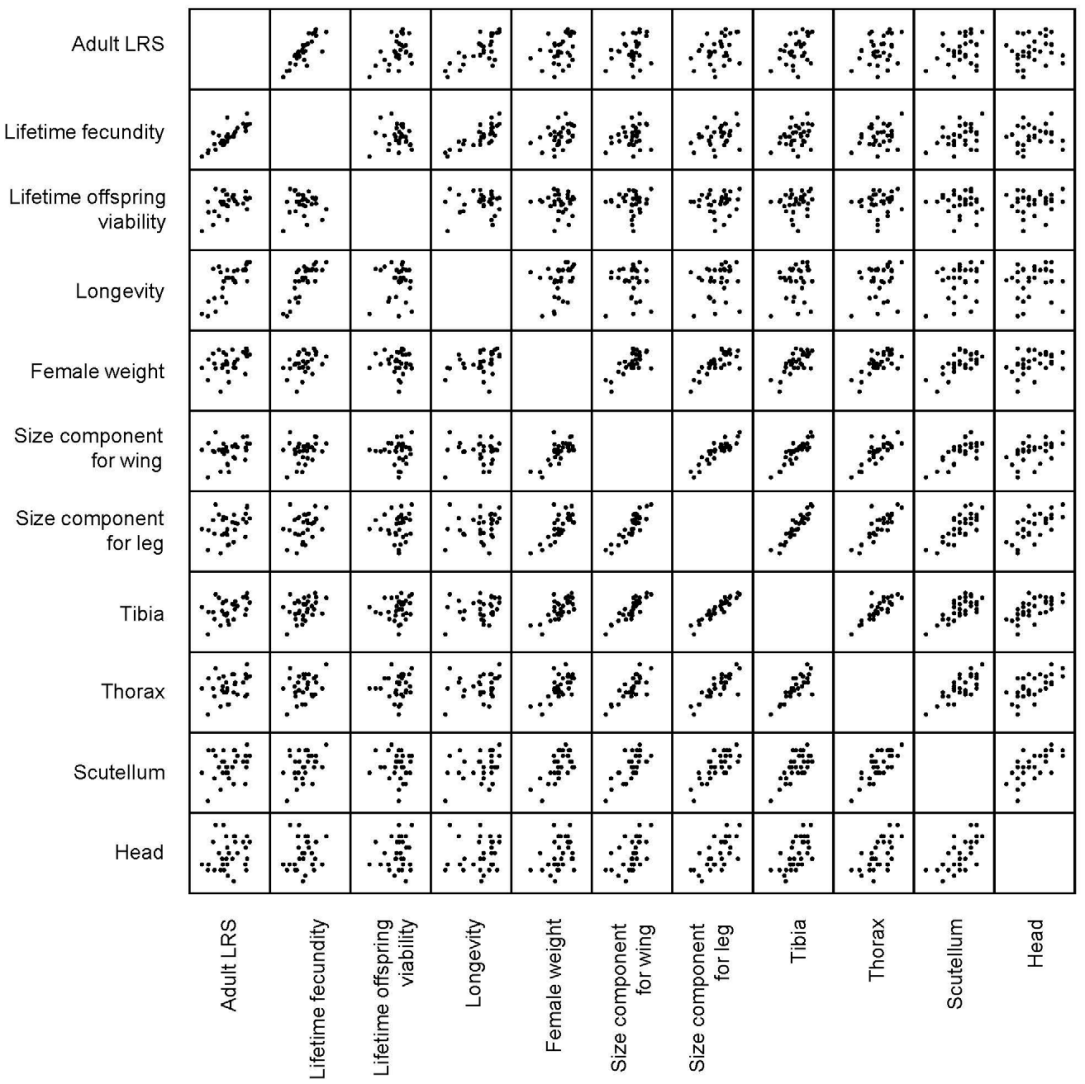
Scatterplots of adult LRS, lifetime fitness components, and size measures. Data is shown only for individuals to whom measurements for all the variables were available.

Phenotypic correlations between the adult LRS measures, fitness components measured over the lifetime of the females, and size measures, together with means and standard deviations of the variables, are shown in Table 1. Figure 5 displays correlations of the variables graphically (not shown for the adjusted LRS measures). From the fitness components measured over the lifetime of the females, fecundity had the highest correlation with adult LRS (r = 0.81). Female longevity and offspring viability were also relatively highly correlated with adult LRS (r = 0.63, and r = 0.51, respectively). Longevity and fecundity correlated positively with each other, but offspring viability correlated with neither longevity nor fecundity. Size measures had modest to weak correlations with adult LRS. Leg size, based on measurements of all segments of the hind legs, had the highest correlation (r = 0.38), followed by female weight (r = 0.32).

**Table 1:** Correlations between adult LRS measures, lifetime fitness components, and size measures, with means and standard deviations of the variables.

	Adult LRS	Adult LRS with 2% mortality	Adult LRS with 4% mortality	Adult LRS with 6% mortality	Adult LRS with 8% mortality	Adult LRS with 10% mortality	Adult LRS with 12% mortality	Lifetime fecundity	Lifetime offspring viability	Longevity (parametric)	Longevity (non-parametric)	Female weight (mg)	Wing size	Leg size (mm)	Tibia (mm)	Thorax (mm)	Scutellum (mm)	Head (mm)
Mean; SD	1027; 48	538; 193	323; 110	214; 75	152; 56	113; 45	88; 37	1689; 654	.61; .16	86; 25	86; 25	3.33; .40	--	--	.97; .04	1.54; .08	.45; .03	.53; .03
Adult LRS		.94** (77)	.80** (77)	.66** (77)	.55** (77)	.48** (77)	.42** (77)	.81** (77)	.51** (73)	.63** (77)	.55** (77)	.32** (77)	.24 (47)	.38* (48)	.24 (70)	.22 (74)	.24 (74)	.12 (75)
Adult LRS with 2% mortality			.95** (77)	.86** (77)	.78** (77)	.71** (77)	.65** (77)	.78** (77)	.51** (73)	.55** (77)	.43** (77)	.36** (77)	.32* (47)	.42** (48)	.28* (70)	.26* (74)	.25* (74)	.17 (75)
Adult LRS with 4% mortality				.97** (77)	.92** (77)	.87** (77)	.83** (77)	.67** (77)	.44** (73)	.44** (77)	.31** (77)	.36** (77)	.38* (47)	.44** (48)	.29* (70)	.28* (74)	.24 (74)	.20 (75)
Adult LRS with 6% mortality					.99** (77)	.96** (77)	.93** (77)	.56** (77)	.37** (73)	.33** (77)	.23* (77)	.36** (77)	.41** (47)	.44** (48)	.28* (70)	.28* (74)	.22 (74)	.22 (75)
Adult LRS with 8% mortality						.99** (77)	.98** (77)	.47** (77)	.30* (73)	.25* (77)	.16 (77)	.31* (77)	.42** (47)	.44** (48)	.25 (70)	.27* (74)	.21 (74)	.23 (75)
Adult LRS with 10% mortality							.99** (77)	.41** (77)	.25* (73)	.20 (77)	.18 (77)	.28* (77)	.43** (47)	.42** (48)	.23 (70)	.26* (74)	.20 (74)	.23 (75)
Adult LRS with 12% mortality								.36** (77)	.22 (73)	.16 (77)	.07 (77)	.26* (77)	.43** (47)	.41** (48)	.20 (70)	.26* (74)	.21 (74)	.23 (75)
Lifetime fecundity									−.05 (73)	.78** (77)	.71** (77)	.30* (77)	.26 (47)	.30 (48)	.17 (70)	.12 (74)	.17 (74)	.10 (75)
Lifetime offspring viability										−.02 (73)	−.06 (73)	.11 (73)	−.05 (45)	.20 (44)	.12 (66)	.18 (70)	.08 (70)	.12 (71)
Longevity (days; parametric)											--	.20 (77)	.00 (47)	.11 (48)	.06 (70)	.03 (74)	.07 (74)	.08 (75)
Longevity (days; non-parametric)												.16 (77)	−.03 (47)	.13 (48)	.01 (70)	.01 (74)	.02 (74)	.16 (75)
Female weight (mg)													.67** (47)	.73** (48)	.64** (70)	.71** (74)	.57** (74)	.38** (75)
Size component for wing														.88** (34)	.86** (46)	.78** (46)	.73** (46)	.47** (47)
Size component for leg															.92** (48)	.80** (47)	.68** (47)	.58** (48)
Tibia																.77** (69)	.61** (69)	.54** (70)
Thorax																	.71** (74)	.48** (74)
Scutellum																		.45** (74)

** significant at the 0.01 level; * significant at the 0.05 level; significance levels are adjusted by Benjamini & Hochberg correction for false discovery rate.

Pearson's correlation coefficients (sample size in parentheses) between the adult LRS measures, lifetime fecundity, lifetime offspring viability, longevity (also non-parametric results shown), and the size measures of the females. On the uppermost row mean and standard deviation of the variables.

**Figure 6:**
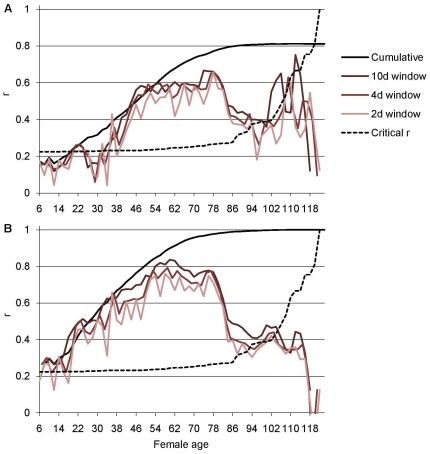
Correlations between adult LRS and cumulative and short-time fecundity and offspring production. Pearson's correlation coefficients (r) between adult LRS and A) cumulative fecundity, and fecundity in sliding windows of 2 days, 4 days, and 10 days, and B) cumulative offspring production, and offspring production in sliding windows of 2 days, 4 days, and 10 days. Above critical r (dashed line) correlations are significant at α = 0.05 level (two-tailed; note that the critical effect size for significance increases with increasing female age because of decreasing sample size). Female age is scored according to the midpoint of the time frame in question.

Correlations between adult LRS and cumulative and short-time measures of fecundity and offspring production are shown in Figure 6. Correlations of the short-time measures of fecundity and offspring production with adult LRS were highly dependent on the time of measurement: for young females the correlations were low, but when measured from older females, the correlations were much higher (up to 0.67 for short-time fecundity and 0.83 for short-time offspring production). For both short-time fecundity and short-time offspring production the highest correlations with adult LRS were reached when the female age was about 50 to 80 days. The length of the time frame had only a minor effect: the correlation of the 10-day measure with adult LRS was generally only slightly higher than that of the 2-day measure. The short-time measures performed well in comparison to the cumulative measures of fecundity and offspring production.

**Figure 7:**
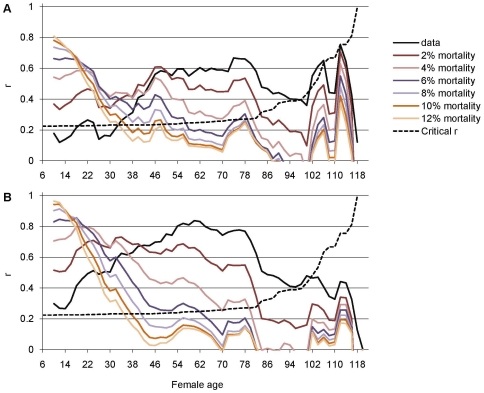
Effect of additional mortality on correlations between adult LRS and 10-day measures of fecundity and offspring production. Pearson's correlation coefficients (r) between adult LRS measures (unadjusted adult LRS, and adult LRS adjusted for additional daily mortality risk of 2, 4, 6, 8, 10, and 12%) and A) fecundity in sliding windows of 10 days, and B) offspring production in sliding windows of 10 days. Above critical r (dashed line) correlations are significant at α = 0.05 level (two-tailed; note that the critical effect size for significance increases with increasing female age because of decreasing sample size). Female age is scored according to the midpoint of the time frame in question.

Correlations between the adjusted adult LRS measures and 10-day measures of fecundity and offspring production are shown in Figure 7. As expected, correlations between the short-time measures of fecundity and offspring production with adjusted adult LRS were generally higher when measured from younger than when measured from older flies; the effect was more pronounced with higher levels of additional mortality risk. Adjusting adult LRS with additional mortality risk also increased variation between the different lengths of time frames: the 10-day measure outperformed the shorter time frames by giving more consistent correlations (data from shorter windows is not shown).

Mortality-adjustment to adult LRS did not have a strong effect on the correlations with size measures (Table 1). If anything, the correlations of size measures were stronger with the adjusted adult LRS measures than with unadjusted adult LRS. This effect was due to generally higher correlation of size measures with early fecundity and offspring production than with late fecundity and offspring production (analysis not shown).

## Discussion

We explored phenotypic correlations between adult LRS, measured as the total number of offspring produced over the adult lifetime of individual *D. littoralis* females in laboratory, and various morphological and life history traits commonly used as fitness surrogates. As could be expected, the lifetime measures of fecundity, longevity, and offspring viability were all relatively highly correlated with adult LRS. Previous research on correlations between adult LRS and other fitness surrogates is rather scarce. However, strong positive correlation between longevity and adult LRS has been documented also in *D. melanogaster* [24] and in the house fly (*Musca domestica*) [25] in laboratory and in some bird [26]–[28] and mammal species [29], [30] in the field. In the housefly [25], the song sparrow (*Melospiza melodia*) [26], and the house martin (*Delichon urbica*) [27], strong correlation was also found between lifetime fecundity and total number of offspring produced.

Correlation of the short-time measures of fecundity and offspring production with adult LRS depended greatly on the time of measurement: when measurements were from older rather than from younger females correlations were surprisingly high. The short-time measures performed well also in comparison to the cumulative measures of fecundity and offspring production. It seems that, if timed correctly, the more practical short-time measures could give as good estimates of adult LRS as can more laborious and time-consuming cumulative measurements. In contrast to our findings, Reed and Bryant [25], exploring the relationship between adult LRS and seven other fitness surrogates in pairs of the housefly, ended up recommending only fitness surrogates covering the entire lifetime of the organism. However, the argument of Reed and Bryant [25] is based on the weak performance of three fitness surrogates measured at the very beginning of the reproductive lifetime of the housefly pairs (age at first reproduction, the size of the first egg clutch, and egg-to-adult viability of the first clutch). We measured short-time fecundity and offspring production of individual females throughout the female lifetime and, as said, discovered that when measured from individuals well into their reproductive life, short-time measures predicted adult LRS surprisingly well. Measuring fitness surrogates from older individuals is of course justifiable only when mortality is negligible; if mortality is high, the older age-classes comprise only a selected subset of the population.

Correlation between adult LRS and short-time components of fitness may depend greatly on the short-time measure used. In the song sparrow, a strong correlation was found between the number of young raised in the first breeding year and total number of young reared by females in their lifetime (r = 0.82) [26]. However, correlation between the number of eggs laid in the first breeding year and the total number of young reared was relatively poor (r = 0.32) [26].

Correlations between adult LRS and size measures were generally weaker than those between adult LRS and measures of life history traits (longevity, lifetime or short-time fecundity, short-time offspring production, and lifetime egg-to-adult viability of offspring). However, two of the size measures correlated reasonably well with adult LRS: leg size and female weight. In fact, by simply weighing the female one can get a better estimate for adult LRS than with an unfavorably timed measurement of fecundity. Tibia length, a commonly used size measure [14], [31], [32], had a lower correlation with adult LRS than the size measure combining all leg segments.

There seems to be a lot of variation in how size measures relate to offspring production between different species studied. Partridge *et al.* [23] documented a strong positive correlation between thorax length and adult LRS in *D. melanogaster* (r = 0.67). This correlation is much stronger than what was found in our study (r = 0.22), in spite of the similar study systems. Strong correlations between offspring production and weight have been documented e.g. in red squirrels (*Sciurus vulgaris*) [33] and in a monogamous rodent (*Peromyscus californicus*) [34]. Scott [35] studied these relationships in Bewick's swans (*Cygnus columbianus bewickii*), and found only moderate to weak correlations between total number of young and female weight and morphological measures. In the house martin, body mass, keel length, and wing length were all very poor indicators of total young reared [27].

In addition to the adult LRS realized in laboratory conditions, we used adjusted measures with additional daily mortality risk of the females. Thus, the adult LRS measures adjusted for mortality weight early reproduction more than later reproduction, and therefore more closely reflect fitness in natural conditions where the flies have evolved. It is well known that predation and other hazards in nature result in shorter lifespan in nature than in laboratory [20], and that mortality caused by predation affects the evolution of life-histories [36], [37]. Estimates of daily mortality risk in natural populations of various *Drosophila* species range from 15% to 55% [20]. Thus, although a lifetime in *D. littoralis* is somewhat longer than in the species used in these studies, the daily mortality estimates used here (2, 4, 6, 8, 10, and 12%, in addition to natural death of the females in the experiment) can be considered conservative. Predictably, and in contrast to what was found for unadjusted adult LRS, the short-time measures of fecundity and offspring production correlated better with mortality-adjusted adult LRS if measured from younger flies than if measured from older flies. Thus, assuming additional mortality risk in nature changes the optimal time frame for short-time measurements of fecundity and offspring production. Interestingly, size measures tended to correlate more strongly with adjusted adult LRS than with unadjusted adult LRS, suggesting that size might predict fitness better in environments where mortality rates are higher.

Because adult LRS combines several fitness components, it is likely to be closely related to the total fitness of individuals. Using adult LRS as a surrogate for total fitness is not, however, totally unambiguous. The number of adult offspring eclosing from the eggs laid by a female is not solely the property of the female, but also that of the offspring themselves, as the offspring have unique genotypes different from their mother. Assigning offspring fitness to the mother may thus lead to erroneous conclusions, especially if the impact of offspring genotype on offspring viability is large in comparison to maternal effects [38]. In this light, lifetime fecundity might be considered a better estimate of female fitness than lifetime offspring production, as fecundity can more clearly be considered a property of the female itself. While achieving consensus on the best fitness measure (total number of eggs vs. total number of adult offspring) is beyond the scope of the current paper, we argue that researchers should always carefully consider how they define individual fitness.

The possible effects of competition are excluded in our study, as the availability of food was not a limiting factor, and only one male and one female fly were introduced to each other. Competition over resources such as food and shelter may not be strong in the natural habitat of the flies, as the population density seemed low at the Tourujoki River area (personal observation). However, other evolutionary processes such as sexual selection might potentially contribute to the reproductive success of the flies [39]. A recent study showed that increased exposure to males changes rate-sensitive fitness estimates of females in *D. melanogaster*, and the direction of the change depends on whether the population is expanding or declining [7]. The effects of competition can thus be complex and dependent on population dynamics.

In summary, the best surrogates for adult LRS of *D. littoralis* females in this study were lifetime fecundity and well-timed short-time measures of fecundity and offspring production. The great variation found in the strength of the relationship between adult LRS and the other surrogates of fitness shows the importance of careful choice of fitness surrogates in empirical research. With short-time measures, it is crucial to pay attention to the timing of the measurements.
